# Economic Evaluation of Individual School Closure Strategies: The Hong Kong 2009 H1N1 Pandemic

**DOI:** 10.1371/journal.pone.0147052

**Published:** 2016-01-28

**Authors:** Zoie Shui-Yee Wong, David Goldsman, Kwok-Leung Tsui

**Affiliations:** 1 School of Public Health and Community Medicine, The University of New South Wales, Sydney, New South Wales, Australia; 2 School of Industrial and Systems Engineering, Georgia Institute of Technology, Atlanta, Georgia, United States of America; 3 Department of Systems Engineering and Engineering Management, City University of Hong Kong, Hong Kong, China; Uniformed Services University of Health Sciences, UNITED STATES

## Abstract

**Background:**

School closures as a means of containing the spread of disease have received considerable attention from the public health community. Although they have been implemented during previous pandemics, the epidemiological and economic effects of the closure of individual schools remain unclear.

**Methodology:**

This study used data from the 2009 H1N1 pandemic in Hong Kong to develop a simulation model of an influenza pandemic with a localised population structure to provide scientific justifications for and economic evaluations of individual-level school closure strategies.

**Findings:**

The estimated cost of the study’s baseline scenario was USD330 million. We found that the individual school closure strategies that involved all types of schools and those that used a lower threshold to trigger school closures had the best performance. The best scenario resulted in an 80% decrease in the number of cases (i.e., prevention of about 830,000 cases), and the cost per case prevented by this intervention was USD1,145; thus, the total cost was USD1.28 billion.

**Conclusion:**

This study predicts the effects of individual school closure strategies on the 2009 H1N1 pandemic in Hong Kong. Further research could determine optimal strategies that combine various system-wide and district-wide school closures with individual school triggers across types of schools. The effects of different closure triggers at different phases of a pandemic should also be examined.

## Introduction

Non-pharmaceutical interventions are practical methods for containing the spread of an emerging infectious disease; they are especially important during the early stages of a pandemic, as antivirals may not effectively treat new outbreaks and vaccination supplies usually require several months to develop [1]. Among the non-pharmaceutical interventions, school closure has received considerable attention from both policy makers and researchers in the public health community [2]. School closure is part of many national pandemic preparedness plans [3–5] and has been heavily implemented during previous pandemics [6–8].

Previous studies have revealed that school closures are usually most effective when they result in a large reduction in contacts, when there is a low basic reproduction number (i.e., disease transmissibility is low), and when attack rates in children are higher than in adults [9]. Other studies have shown that school closure is an effective social distancing strategy [10–12] and that schools played an important role in the transmission of past influenza outbreaks [13, 14], as children are efficient transmitters of influenza, are more vulnerable to infection, and are at higher risk of infection consequences than adults [15, 16]. Lee et al. [17] demonstrated that school closures could delay an epidemic peak and thus allow extra time for medical interventions. A preliminary study of the 2009 H1N1 pandemic in Hong Kong (HK) demonstrated that strategies involving school closure interventions outperformed certain medical interventions [18].

On the other hand, school closures also inevitably cause social disruptions and incur substantially higher costs than the unmitigated baseline [19], especially if the closure period is long. Halder et al. [20] suggested that a school closure of limited duration could be more cost-effective than a continuous lengthy school closure. In any case, determining the most cost-effective and timely strategies to contain pandemics of transmissible diseases such as influenza is of great concern to government policy makers and healthcare professionals. There is a substantial body of research on modelling school closures and devising preparedness plans in the field of infectious disease epidemiology. However, a recent systematic review pointed out that there is a lack of simulation models that compare the effectiveness of individual, regional, and national school closure strategies [9].

Modelling population dynamics at the more-detailed community level opens up opportunities to examine the effects of regional-level intervention strategies. Our study uses data drawn from the 2009 influenza pandemic in HK to evaluate the cost-effectiveness of various school closure strategies, particularly the closure of individual schools.

## Methods

### School activities in the 2009 Hong Kong pandemic

The 2009 HK influenza H1N1 pandemic has provided us with great opportunities to understand the disease’s epidemiology and the effectiveness of a range of interventions on containing outbreaks [21–26]. Previous studies recorded the number of laboratory-confirmed H1N1 cases by date of illness onset. The various control measures used and their durations have also been reported [26]. The first indexed case, which was also the first confirmed case in Asia, was found on 1 May 2009 and the first indigenous transmission was confirmed on 10 June 2009 [2]. Subsequently, there was a quick change from a “containment phase” to a “mitigation phase”, which sought to effectively implement non-pharmaceutical interventions. The government implemented school closures throughout the pandemic period. For instance, a two-week system-wide proactive closure of kindergartens and primary schools was implemented on 11 Jun 2009 [22]. Two individual secondary schools at Kwun Tong and Causeway Bay were closed for two weeks on 28 May 2009 and 10 Jun 2009 due to confirmed cases at these schools[27, 28]. Although secondary school summer vacation started at the end of June and ended at the end of August, school activities including summer classes and extra-curricular activities continued throughout the period. During the summer, there were reports of outbreaks of human swine H1N1 influenza in schools, and the government had to suspend classes at a secondary school in the Southern District on 26 Aug for one week [29]. A previous study using a parsimonious transmission model demonstrated a 25% reduction in the original transmission due to these actions [22]. As these school suspensions were sporadic and other various intervention strategies were implemented throughout the outbreak (such as public health campaigns, medical resource mobilisation, and antiviral treatment of confirmed cases), the precise effects of these school closures are still unclear. Our goal was to prepare for the next pandemic by estimating the health and economic effects of different systematic individual school closure strategies and trigger thresholds.

### Age-stratified region-specific disease-spread simulation model

We developed an age-structured region-specific Susceptible-Exposed-Infected-Removed (SEIR) compartmental model [30] to mimic pandemic influenza transmission based on a synthetic population generator [31] using data for Hong Kong from the Census and Statistics Department [32]. The key characteristic of the model was that it considered the disparity of demographic structures and regional dynamics across districts, instead of assuming a homogeneous individual contact structure. We specified district-specific distributions of age, household composition, workplaces, and schools. In addition, population mobility at the district level was specified, allowing for cross-community activities among both students and workers across HK. School sizes and student demographics at various school levels by districts were stratified based on regional statistics. This dataset enabled us to examine localised school closure strategies; for example, we could consider the effects of the closure of an individual school in one district using a variety of regional triggers or thresholds. We calibrated the model using the age-stratified estimated attack rates provided by Wu et al. [23], which reflect the effects of the fragmentary school closures in the early stages of the outbreak.

### Closure of individual schools

We modelled various combinations of school closure strategies, and for each we examined the effects of school types closed, closure modes, closure triggers, and closure length. The simulation model ran various system-wide, district-wide, and individual-based school closure strategies and was set to close schools of various levels (i.e., kindergarten (K), kindergarten and primary (KP), all schools types including secondary (KPS), or any combination of those). The detailed study outcomes for the system-wide and district-wide school closures have been reported previously [30]. The current study focuses on the cost analysis of individual-based school closure strategies with various closure triggers and lengths. Here, individual school closure refers to the shutting down of a single school of a pre-defined school type after the number of confirmed cases reaches the selected threshold level.

We attempted to incorporate realistic scenarios that would be feasible, potentially implementable, and practical into our school suspension strategies. According to a document discussing Hong Kong’s preparedness for influenza pandemic prevention and protection, the closure of schools, as a social distancing measure, may be instituted to reduce population mobility and related social contacts [4]. Although the practice of school closure may vary depending on a disease’s transmission routes, infectivity, incubation, infectious period, and scale of outbreak, most systematically instituted school closures involve/begin with younger children [22], specifically, kindergarten children and sometimes children in primary schools. When the government makes the decision to close schools, the government and public tend to first protect the younger children, i.e., those who are highly susceptible and less aware of health conditions. Young children are generally perceived as a more-vulnerable group in a society. Although they constitute a relatively small proportion of the population, they receive more attention and have close contact with their parents/family members. School suspension for this group may not have a significantly disproportionate effect on the overall containment of the outbreak, but their protection has a certain symbolic meaning within public health.

This study was not restricted to kindergarten closures, but also considered systematic school closure strategies that included other junior groups. In any case, kindergarten groups were considered in all of the individual-level systematic school closure scenarios. For example, any school closure trigger that applies to secondary schools will always also apply to primary schools and kindergartens. In our experiments, the school closure trigger was a certain **n**umber of **c**onfirmed cases at a **p**articular school (NCP): 1, 3, 5, 10, and 20. In the past, school closures have lasted from 1 week to several months [2]. Therefore, **s**chool **c**losure **l**engths (SCL) of 1, 2, 3, 4, 6, 8, 12, and 16 weeks were considered in this study. We ran each simulation setting 20 times to obtain low-variance estimates of the various quantities of interest; the simulated outcomes were collected and analysed together. System-wide school closure includes those scenarios with a closure trigger based on the **f**irst **c**ase is **f**ound (FCF) and the overall **n**umber of **c**onfirmed cases in the community (NC). District-level school closure covers those strategies when the **n**umber of **c**onfirmed cases in the **d**istrict reaches a threshold level (NDC), and individual based school closure covers those scenarios with a trigger based on a certain **n**umber of **c**onfirmed cases at a **p**articular school (NCP). For the remainder of the paper, we have denoted various combinations of school closure settings systematically. For instance, NCP5-KP-SCL1 refers to NCP with a trigger of 5 for KP school levels and a closure period of one week.

### Economic model

The economic effects of each school closure strategy were evaluated by first establishing an economic model for the pandemic. Following Andradóttir et al. [33], the total cost of each intervention scenario was set as the sum of the medical costs associated with the illness, the costs associated with parents staying at home with sick children, and other costs associated with school teachers, parents, and children staying home due to school closures. The cost incurred for the baseline was also estimated using this method. The medical costs associated with illness include outpatient visits, prescription and over-the-counter drugs, hospitalisation, and lost earnings due to death. In every simulation run we counted the number of infected people in each age group, the number of parents staying home with sick students, and the number of people staying home due to school closure. Table 1 gives the cost estimates and medical price indices drawn from various sources in Hong Kong [34–38] and global studies [33, 39, 40]. The detailed cost parameter citations and calculations are provided (Tables A and B of S1 File).

**Table 1:** Cost analysis model parameters (HKD). Cost estimates are from [33–40], as appropriate.

Outcome Category Item	Children (0–18)	Adults (19–59)	Seniors 60+)
**Outpatient Visits**			
Average no. visits per case [33, 39]	1.52	1.52	1.52
Net payment per visit [33, 35, 36, 39]	1,391	1,079	1,419
Average co-payment per visit [34]	100	100	100
Net payment per prescription [33, 39, 41]	10.8	15.5	15.5
Average co-payment per prescription [33, 39, 41]	106.9	153.9	153.9
Average prescriptions per visit [33, 39]	0.9	1.8	1.4
Days lost [33, 39]	3	2	5
Value of one day lost [33, 37, 42]	585.2	638.4	585.2
Subtotal	4,202.2	3,329.1	5,494.5
**Hospitalisation**			
Hospital costs [33, 39, 41]	9,812	20,017	32,352
Net payment per outpatient visit [33, 39]	2,100	2,669	2,895
Average co-payment for outpatient visit [34]	100	100	100
Most likely no. of days lost [33, 39]	5	8	10
Value of one day lost [33, 37, 39, 42]	585.2	638.4	585.2
Subtotal	14,938	27,893.2	41,199
**Deaths**			
Average age [33, 39]	9	35	74
Present value of earnings lost [33, 38–40]	2,721,253	2,779,026	176,319
Most likely hospital costs [33, 39]	11,480	25,304	39,209
Subtotal	2,732,733	2,804,330	215,528
**Ill but no medical care sought**			
Days lost [33, 39]	3	2	5
Over-the-counter drugs [33, 39, 42, 43]	71.9	71.9	71.9
Value of one day lost [33, 37, 39, 41]	585.2	638.4	585.2
Subtotal	1,827.5	1,348.7	2,997.9
**Staying at home**			
Value of one day lost [37]	585.2	638.4	585.2

According to Wu et al. [23], the case-hospitalisation rates in the 2009 influenza pandemic ranged from 0.47% to 0.87% among people aged 5 to 59 years; the age-group-specific case-fatality rates were from 0.4 to 26.5 cases per 100,000 infections in people aged 5 to 59 years. Combining this with the cost structure suggested by Meltzer et al. [39] and Andradóttir et al. [33], we obtain the age-stratified healthcare decision model for the Hong Kong outbreak illustrated in Table 2.

**Table 2:** Age-stratified healthcare outcomes model [23, 33, 39].

	Probability of Outpatient Visit	Probability of Hospitalisation (×10^−3^)	Probability of Death (×10^−4^)	Probability of Ill but No Medical Care Sought
Children	0.165	8.100	0.150	0.827
Adults	0.040	6.660	1.200	0.953
Seniors	0.045	8.700	2.650	0.946

### Sensitivity analysis

The effects of the uncertainty in our input parameters and assumptions on the uncertainty in the final cost model outcomes were examined using univariate sensitivity analyses. In particular, we inspected the key input parameters: (i) the ascertainment rate (i.e., the probability of confirmed illness given the number of symptomatic infected persons), and (ii) the conditional probability that sick children will be withdrawn from school within the first three days of the symptomatic period. The ascertainment rate was of concern as this value can alter the effect of school closure triggers on decreasing transmission; and the conditional probability of the withdrawal of sick children may affect the number of undetected infected students in schools, which in turn affects the spread of infection within a school. Moreover, the conditional probability value may be uncertain, as it varies from one school to another due to disparities in control measures for detecting sick children and the experience of individual gatekeepers. Thus, both of these values affect the number of sick children who are in contact with other susceptible children in a school. Possible ranges of values for the conditional probability of withdrawal were tested from 0.2 to 0.5 (for the first day of symptoms), 0.6 to 0.9 (for the second day), and 0.75 to 0.9 (for the third day). Low ascertainment rates were also investigated, ranging from 0.05 to 0.7. We also conducted probabilistic sensitivity analysis based on the uncertainty of cost parameters; the detailed model and results are provided (Figures A to D in S1 File).

## Results

Based on a set of per-contact influenza infection transmission probabilities within contact groups, an overall attack rate of 9.21% [the half length of the corresponding 95% confidence interval (CI) is 0.016%] was achieved for the baseline scenario. The infection attack rates in each age group fell within the 95% CIs of the references values [23]. A basic reproduction number (R_0_) of 1.1 was observed, which is within the estimates of the 2009 H1N1 influenza pandemic [44]. The details of the calibrated baseline model are provided in Wong et al. [30]. In the following subsections, we discuss the simulated outcomes of various individual school closure models.

### Overall attack rates and epidemic curves

**Fig 1:**
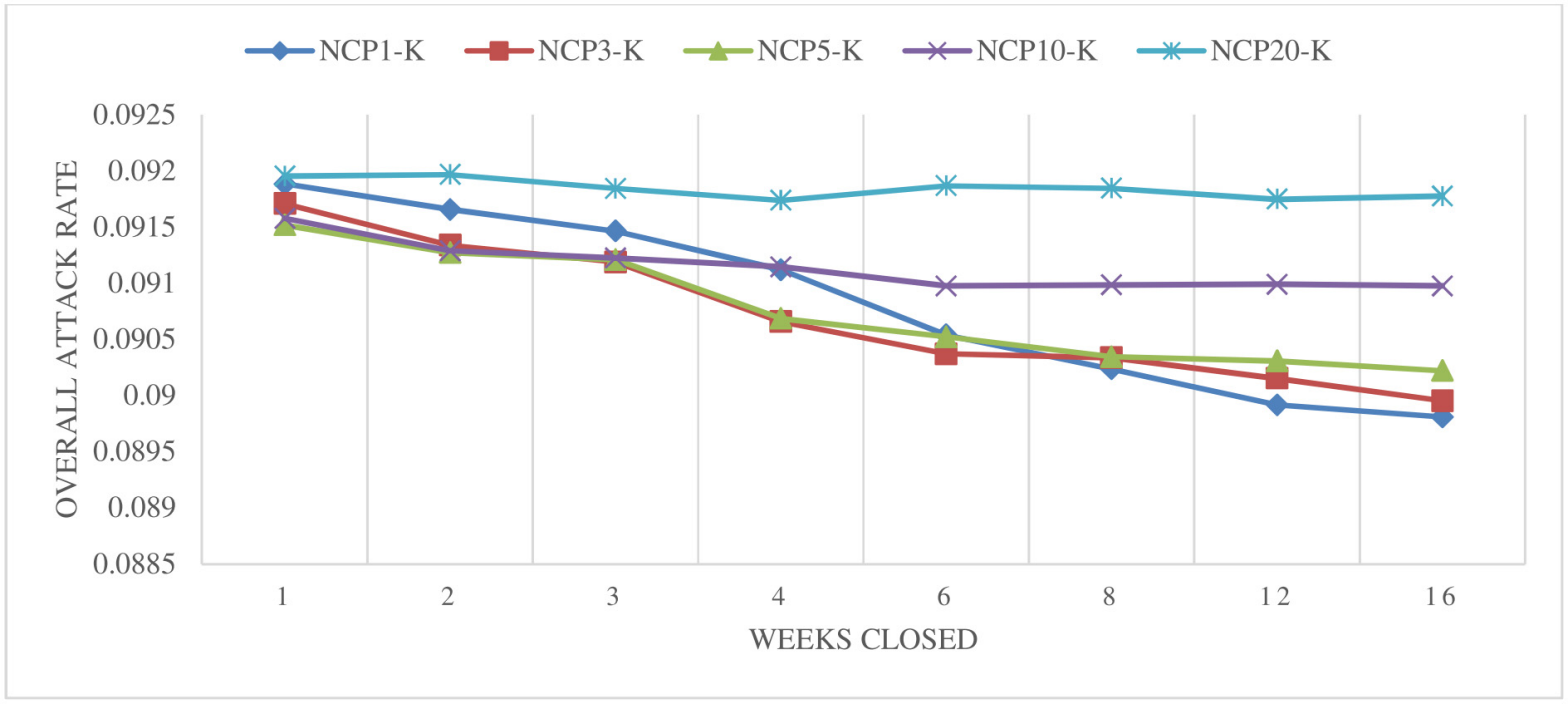
Closing schools based on the number of confirmed cases at a particular school (individual school closure)–Kindergarten.

We first present the simulated overall attack rates for each scenario. Fig 1 shows the simulated overall attack rates for the individual school closure scenarios (kindergarten only) based on the number of confirmed cases at a particular school. For a large trigger (such as 10 and 20), a decreasing overall attack rate was not apparent (the overall attack rate was around 9.10–9.20% across all of the closure lengths). For smaller trigger numbers (such as 1, 3, and 5), the effect was relatively stronger; however, even the best scenario (NCP1-K) only achieved a 0.2% decrease in the overall attack rate, compared to the baseline. We observed that individual school closure may be ineffective if it is only applied at the kindergarten level, perhaps because kindergarten students make up only about 2% of the population (149,171 out of 6,890,476 students). Although closing the schools of this small group does not have a tremendous effect on the overall effort to contain the outbreak, this group may be most vulnerable to infectious disease and thus such actions may be part of a strategy to protect younger children.

**Fig 2:**
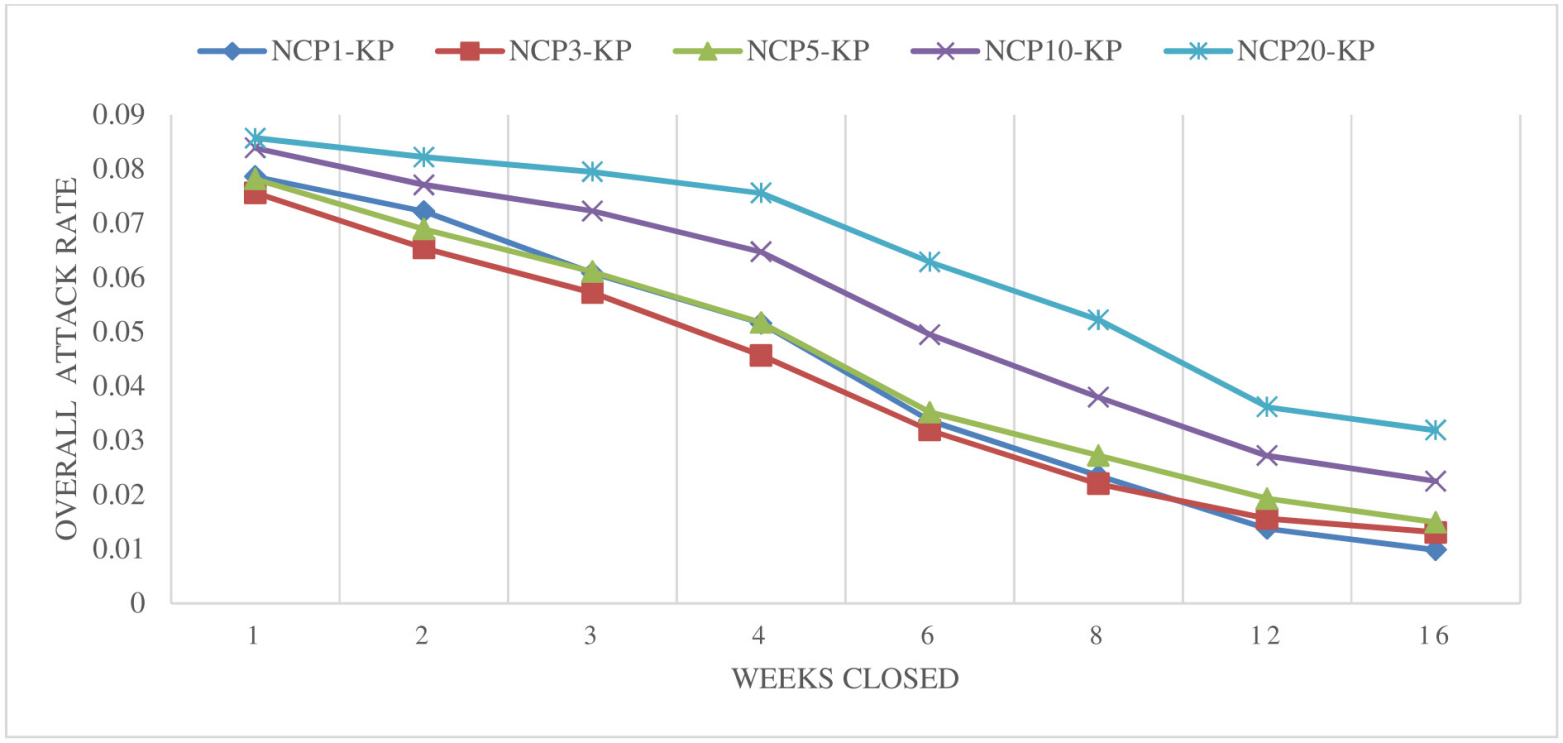
Closing schools based on the number of confirmed cases at a particular school (individual school closure)–Kindergarten and Primary.

Fig 2 illustrates the estimated changes in overall attack rates for various individual school closure scenarios that are applied to kindergarten and primary schools. Across all of these settings, closing schools for longer periods resulted in greater decreases in the overall H1N1 attack rates. This generally agreed with the results of a preliminary study of system-wide and district-wide school closures [30]. However, the individual school closures scenarios (in terms of decreasing overall attack rates) outperform the system-wide and district-wide closures with similar closure periods. Similar to the results shown in Fig 1, a relatively small trigger number of 1, 3, or 5 cases had a better effect than larger thresholds of 10 or 20. The overall attack rate (with triggers of 1, 3, or 5) ranged from 7.9% to 1.0% across all of the closure lengths (from 1 to 16 weeks). However, a long closure period may not be economically feasible or practical in reality, as it brings about tremendous social disruptions. In the following cost-effectiveness section, we evaluate the financial effects of these scenarios.

**Fig 3:**
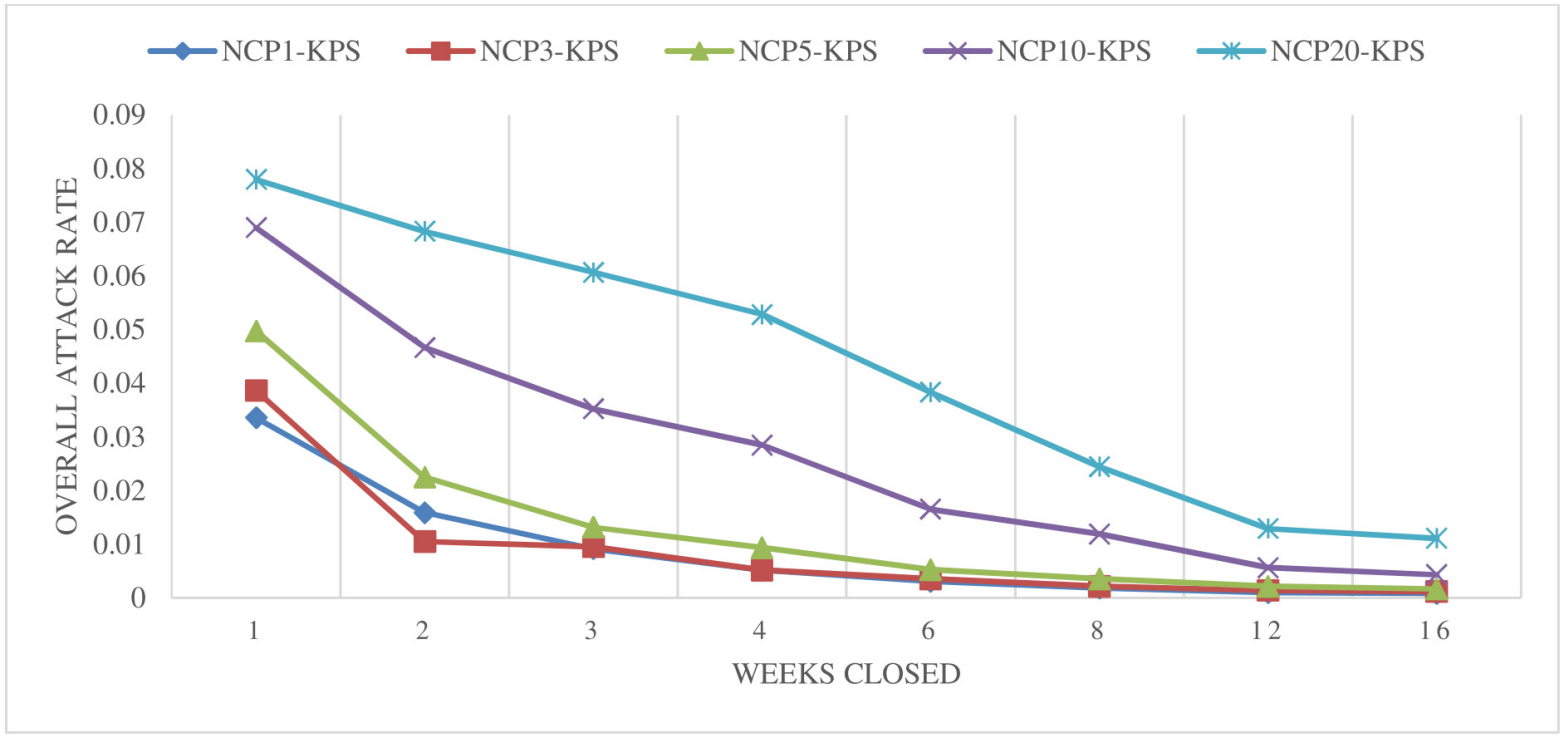
Closing schools based on the number of confirmed cases at a particular school (individual school closure)–All Schools.

Fig 3 illustrates the results of simulated school closure strategies based on the number of confirmed cases at a particular school for all types of schools (i.e., KPS). Individual school closures under this setting showed promising results for effectively slowing down the spread of disease, even for closure periods of only one week. The best scenarios tended to have smaller trigger thresholds (such as 1, 3, or 5). But even scenarios with relatively large thresholds of 10 and 20 achieved better results than the scenarios that considered only KP and K classes. Taking the scenario of NCP3-KPS as an example, the overall attack rate was reduced to less than 4%, even when schools were only closed for one week. This scenario posited that kindergartens, primary, and secondary system schools would close for one week if three confirmed H1N1 cases were detected at a particular school. Note that in this scenario each school was only closed once due to an individual school trigger. Thus, the dates of the school closures for different schools vary according to the number of confirmed cases at the particular schools.

Similar experiments were carried out previously on system-wide and district-wide school closures using different triggers [30]. A school-system closure strategy involves shutting down the entire school system once a certain trigger or threshold is reached. A district-wide closure strategy involves closing all of the schools in a district when the district illness threshold is reached. Compared to these system-wide and district-wide school closure strategies, the individual school closure strategies, in general, achieved much better results in decreasing overall attack rates than the traditional school closure strategies. We also observed that including all types of schools in the scenarios made a difference in the individual school closure results. Given the same trigger and closure length, scenarios that closed all school types (KPS) were typically more successful than scenarios in which only kindergartens and primary schools (KP) were closed; and these KP scenarios performed better than scenarios in which only kindergartens were closed.

**Fig 4:**
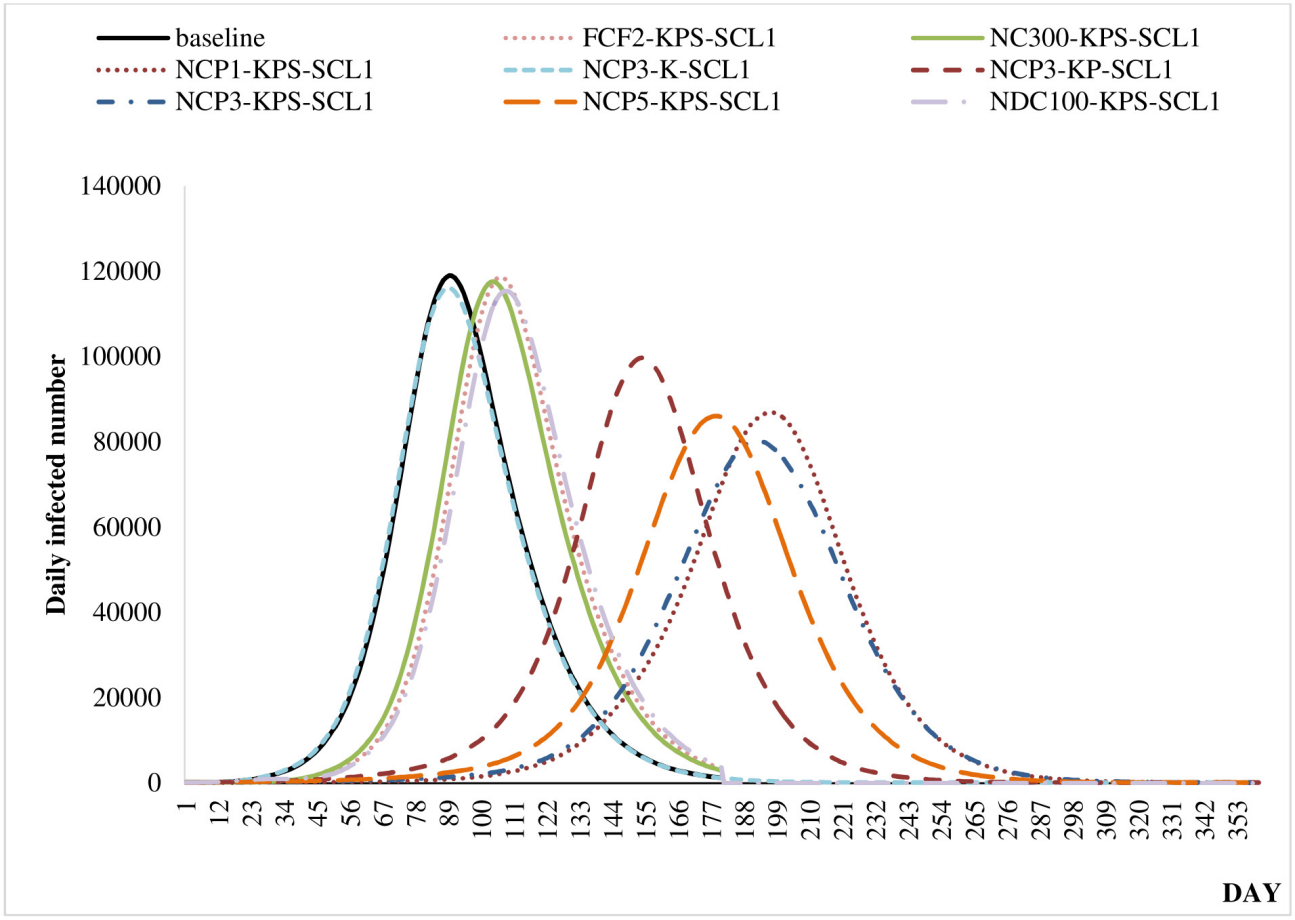
Epidemic peaks of baseline and selected school closure scenarios (closure length: one week).

All of the scenarios with a school closure length of one week were compared using epidemic curves. An epidemic curve illustrates the number of confirmed H1N1 cases over time. We compared the curve of the baseline scenario with those of the mitigation scenarios to quantify the delays in the infectious disease spread. We selected the best-performing individual school closure scenario for each school type—which turned out to be NCP3-K, NCP3-KP, and NCP3-KPS. To highlight the effect of individual school closure strategies, we studied the best scenario based on other school closure strategies [30], including: system-wide school closure after the **f**irst **c**ase is **f**ound with an onset of two weeks (FCF2-KPS); system-wide school closure once the overall **n**umber of **c**onfirmed cases reaches 200 (NC200-KPS); and district-level school closure when the **n**umber of **c**onfirmed cases in the **d**istrict reaches 100 (NDC100-KPS). Our study predicted that closing individual schools would delay the epidemic peak under all of the school closure scenarios. As depicted in Fig 4, for a school closure period of one week (SCL1), various scenarios (including FCF2-KPS, NC300-KPS, and NDC100-KPS) delayed the epidemic peak by two to three weeks compared to the baseline scenario; and the NCP3-KPS scenario outperformed all of the other strategies and achieved a 15-week delay in the epidemic curve. Similar to other school closure studies, our study predicted that the peak would be lower than that of the unmitigated baseline scenario. For instance, the NCP3-KPS scenario (Day 197: 86,961 cases) achieved a 27% decrease in the unmitigated peak compared to that of the baseline scenario (Day 89: 119,038). This result generally agrees with the results of previous prediction studies that found reductions of 20–60% in the peaks [9].

### Cost-effectiveness analysis

A school closure strategy is a non-pharmaceutical intervention that can be implemented at the early phase of a pandemic. It inevitably incurs high costs as it disrupts usual societal practices so that parents may need to stay home to take care of school-age children. As a result, it is important to investigate the cost-effectiveness of different school closure scenarios so that an economically viable strategy that considers the relative costs and outcomes can be devised. Therefore, this study analysed the cost-effectiveness of various individual-based school closure strategies. We included the best scenarios from all of the closure types (including NCP3-K, NCP3-KP, and NCP3-KPS) and compared the results among a variety of individual school closure scenarios. Based on the proposed cost structure discussed in the economic model section, we calculated the cost of each intervention (as well as the baseline case). The cost of the baseline scenario for this city of 6.9 million people was estimated to be HKD2.55 billion (95% CI: +/- HKD4 million) or USD330 million (CI: +/- USD0.5 million); this compares with a baseline cost of about USD75 million for a North American city with a population of 650,000 people [33]. Note that the baseline scenario does not include any school closure costs.

**Fig 5:**
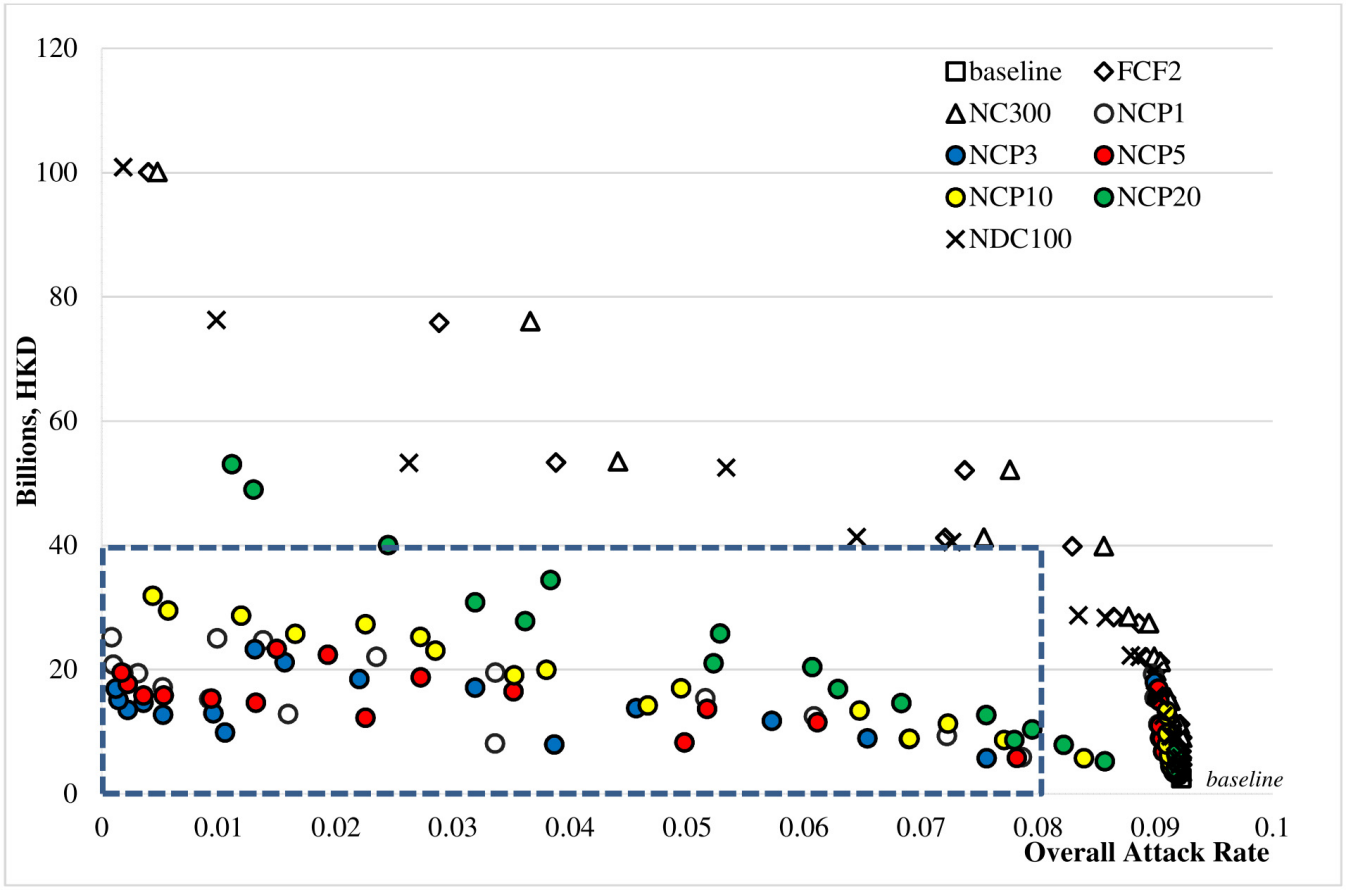
Cost-effectiveness of school closure strategies. Note: Except for the baseline scenario, the other 8 school closure scenarios were run under 3 different school closure modes (K, KP, KPS) and 8 school closure periods (SCL 1, 2, 3, 4, 6, 8, 12, 16). Thus, there are 193 sets of experiments in total, each of which was comprised of 20 replications. The mean cost incurred and the overall attack rate under each set of experiments are displayed.

As shown in Fig 5, individual school closure strategies were more cost-effective than system-wide or district-wide closure strategies in terms of cost-effectiveness. All of the scenarios with overall attack rates less than 8% and intervention costs of less than HKD40 billion (see the blue rectangle in Fig 5) belong to the NCP scenario family (i.e., NCP1, NCP3, NCP5, and NCP10).

### Sensitivity analysis

**Fig 6:**
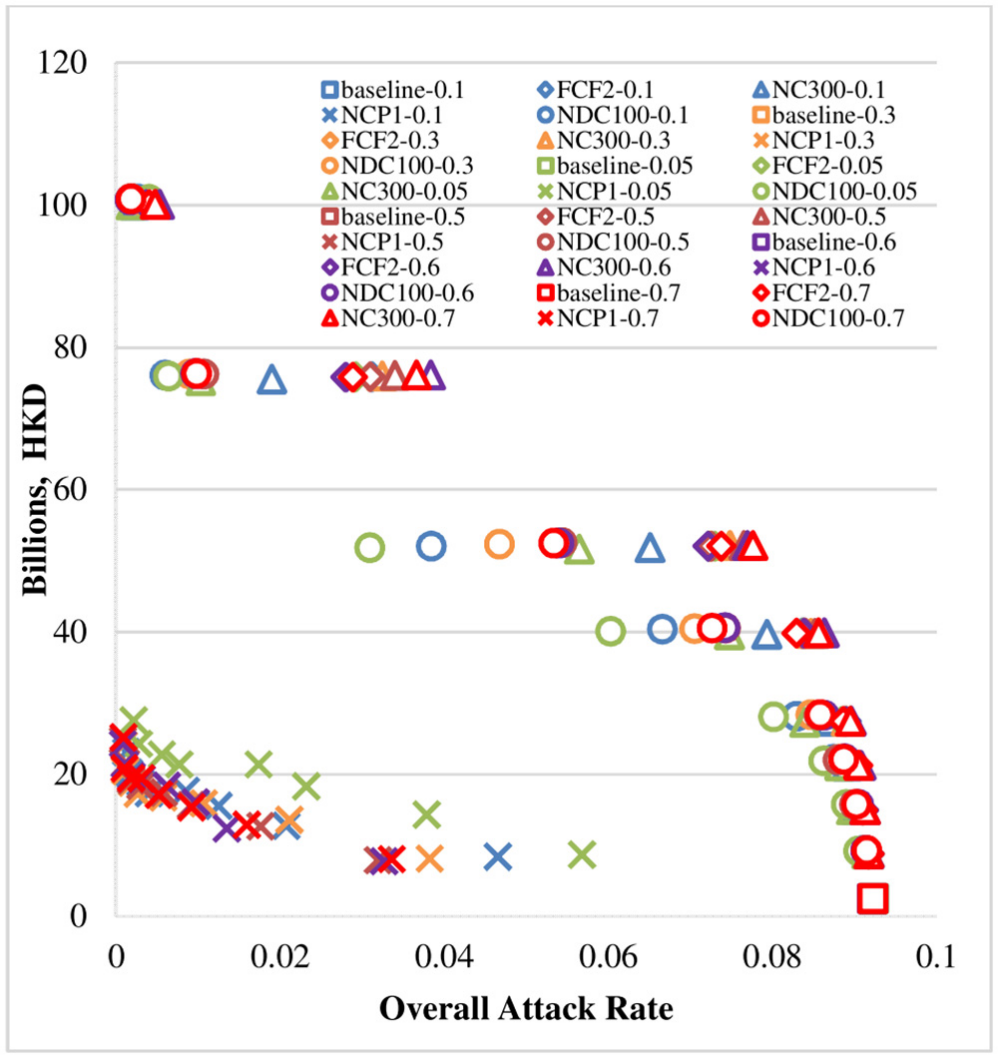
Sensitivity analyses with varying ascertainment rates. Note: Except for the six baseline scenarios, the other 24 school closure scenarios (closure mode: KPS) were run under eight different school closure periods (SCL 1, 2, 3, 4, 6, 8, 12, 16). Thus, there are 198 sets of experiments in total, each of which was comprised of 20 replications. The mean cost incurred and the overall attack rate under each set of experiments are displayed.

**Fig 7:**
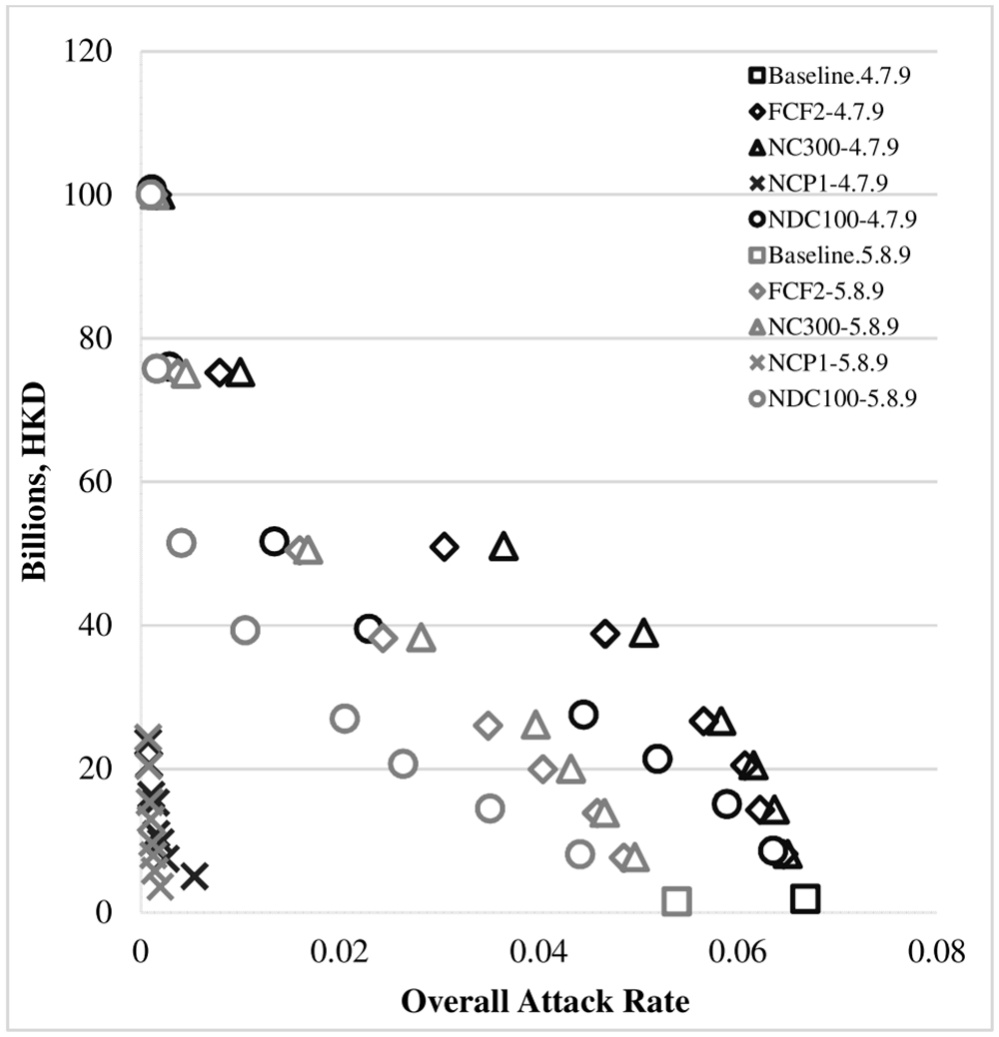
Sensitivity analyses with varying conditional probabilities of withdrawal. Note: Except for the two baseline scenarios, the other eight school closure scenarios (closure mode: KPS) were run under eight school closure periods (SCL 1, 2, 3, 4, 6, 8, 12, 16). Thus, there are 66 sets of experiments in total, each of which was comprised of 20 replications. The mean cost incurred and the overall attack rate under each set of experiments are displayed.

Referring to [33], the baseline values of the conditional probability of withdrawal for kindergarten students were set to 0.3–0.7–0.8 (for days 1, 2, and 3, respectively) and for primary and secondary students to 0.2–0.6–0.75 (for days 1, 2, and 3); and the baseline value of the ascertainment rate was set to 0.7. Figs 6 and 7 illustrate the univariate sensitivity analyses with adjusted input parameters for the ascertainment rate and the conditional probability of withdrawal of sick children during the first three days of the symptomatic period. We examined the possible ranges of values of the conditional probability of withdrawal from 0.2 to 0.5 (for the first day), 0.6 to 0.9 (for the second day) and 0.75 to 0.9 (for the third day). Low ascertainment rates were also investigated, ranging from 0.05 to 0.7. The same cost-effectiveness pattern was found as in the cost-effectiveness analysis section, and the NCP scenarios consistently achieved better cost-effectiveness in terms of cost per case prevented due to the intervention. (See the lower-left-hand corners of both Figs 6 and 7.) This sensitivity analysis and the probabilistic sensitivity analysis evaluating the uncertainty of key cost parameters (S1 File) demonstrated the robustness of the original analyses’ conclusions by examining the effects of our key assumptions in light of uncertainty about the input parameters.

## Discussion

**Fig 8:**
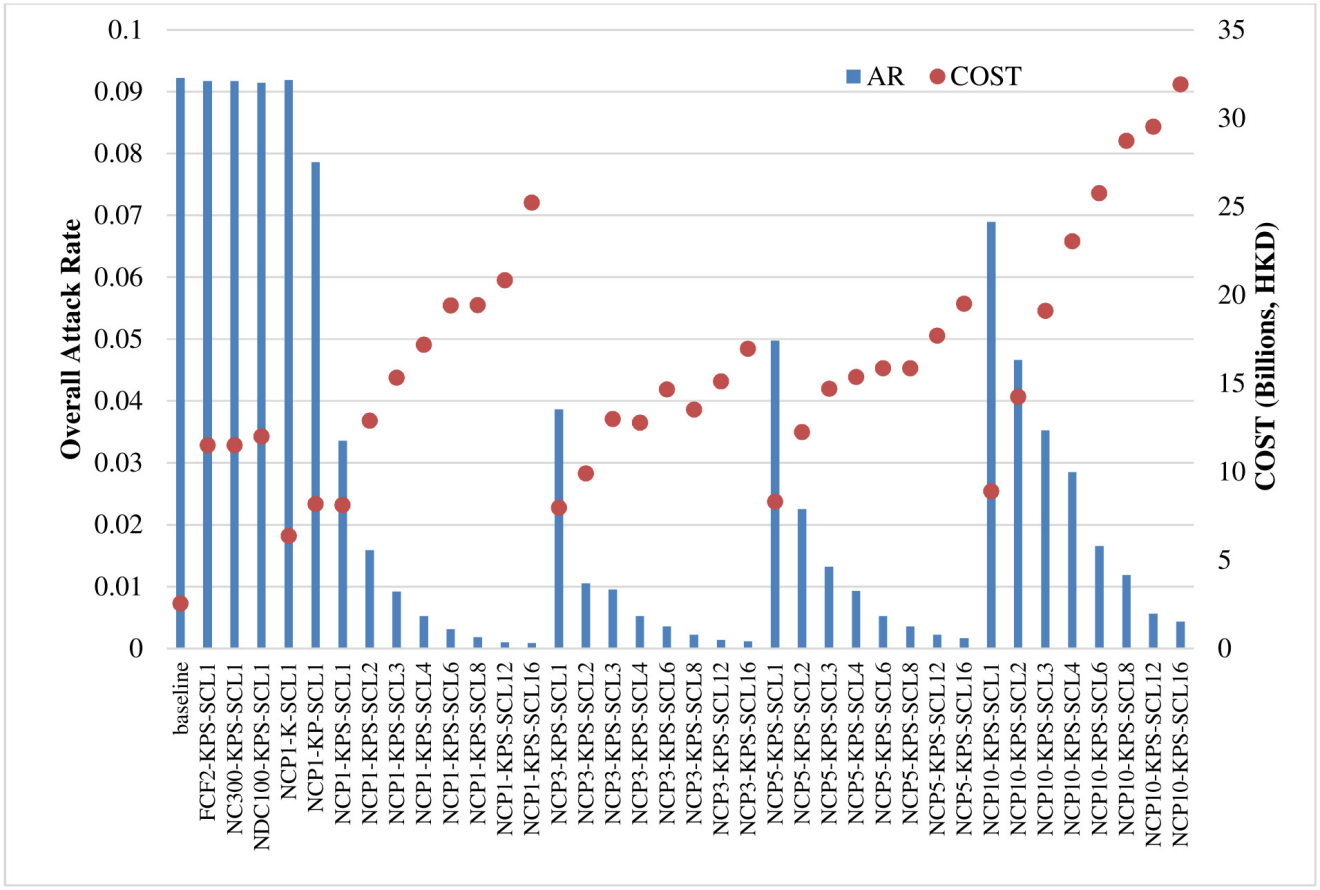
Cost-effectiveness in terms of overall attack rates (ARs) and cost (in billions of HKD).

The above results show that individual school closure strategies appear to effectively hinder the spread of disease. Based on the cost-effectiveness analysis (Fig 5), individual school closure strategies generally performed better than other traditional strategies. Among the individual school closure strategies, those that closed all types of schools and those that had lower trigger thresholds performed better. Fig 8 depicts the cost-effectiveness analysis in terms of overall attack rate and intervention cost in billions (HKD). We compared various individual school closure strategies (NCP1-K-SCL1, NCP1-KP-SCL1, and other full scenarios of NCP-KPS) with the baseline scenario and with selected system-wide (FCF2-KPS-SCL1, NC300-KPS-SCL1) and district-wide (NDC100-KPS-SCL1) strategies.

Compared to the baseline case, the selected system-wide and district-wide cases achieved a meaningful drop in overall attack rates, but incurred high costs. The individual school closure strategies for kindergartens (NCP1-K-SCL1) and kindergartens and primary schools (NCP1-KP-SCL1) had slightly lower costs than individual school closure scenarios that included all school types (such as NCP1-KPS-SCL1), but the resulting overall attack rates of the former two were much higher than for the latter. Thus, closing all types of schools based on individual thresholds was found to be more cost-effective than strategies that closed only kindergartens or only kindergartens and primary schools. As not all school closure lengths decreased overall attack rates, school closure modes and triggers are important when choosing a strategy. Among the individual-based KPS scenarios, closing schools for longer periods of time was more effective, but inevitably more expensive than shorter scenarios. Furthermore, scenarios with small trigger values (such as 1, 3, 5) were more cost effective than other scenarios.

**Fig 9:**
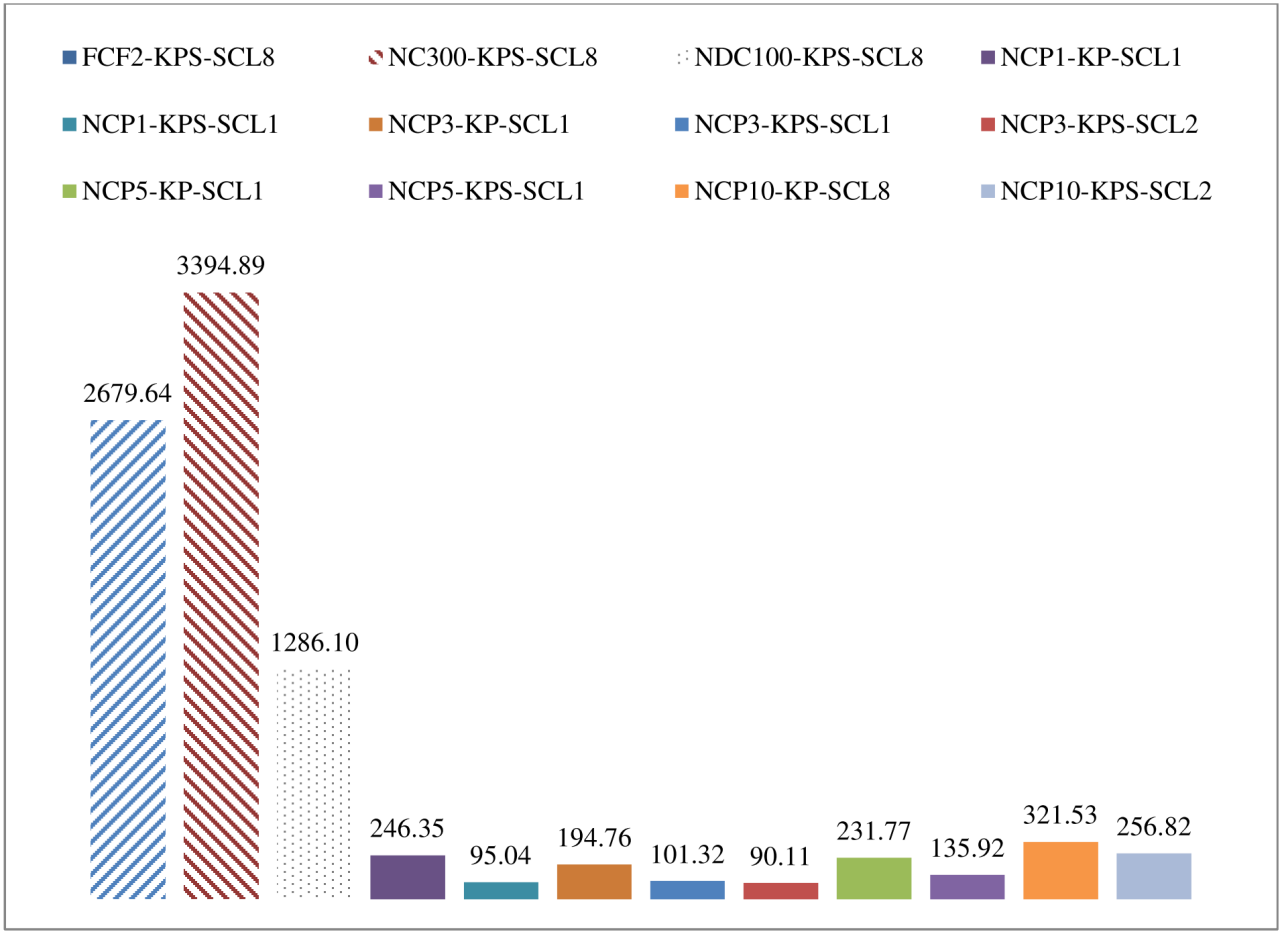
Incremental cost-effectiveness ratios of the best scenarios.

Table 3 displays the breakdown of infected cases by group along with the associated costs and incremental cost-effectiveness ratio (ICER) [45]. We interpret ICER as the cost difference between a particular school closure strategy and the baseline, divided by the net reduction in overall attack rates. Thus, a lower ICER value is associated with a more cost-effective scenario. Fig 9 shows ICER results for the best FCF, NC, NDC, and selected NCP school closure scenarios; and the cost-effectiveness acceptability curves (CEACs) [46–48] of the best scenarios of each school closure setting are given in Figures A to D in S1 File. The expected numbers of deaths from each scenario are also provided in Table 3. This information will be useful for further analysis to calculate other health benefits indicators such as quality adjusted life years (QALYs) gained and disability adjusted life years (DALYs). We also report the total cost per 100,000 population and the cost per case prevented under the best-case scenarios. A previous study [20] suggested that closing school for 2 weeks, 4 weeks, or 8 weeks cost USD1,308, USD1,372, and USD1,868, respectively, for each H1N1 infection case prevented. We found that the cost per case prevented under a school closure scenario can be reduced to less than USD1,200 as soon as any school is closed when that school hits the small threshold. The best three scenarios are NCP3-KPS-SCL2, NCP1-KPS-SCL1, and NCP3-KPS-SCL1; and the estimated costs per case prevented by the interventions were USD1,145, USD1,340, and USD1,255, respectively.

**Table 3:** Breakdown of infected cases, ICER, and costs of the best scenarios.

	# Infected Children (0–18)	# Infected Adults (19–59)	# Infected Seniors (60+)	Overall AR	Change compared to baseline	Medical costs of illness (HKD, bn)	Cost to stay home with sick children (HKD, bn)	Cost to stay home due to school closure (HKD, bn)	Total Cost (HKD, bn)	Total Cost per 100,000 people (USD, m)	ICER (HKD, bn)	Exp # deaths	Cost per Case Prevented (USD)
Baseline	510,459	402,419	34,765	9.22%		2.11	0.43	0.00	2.55	4.64		65.08	-
FCF2-KPS-SCL8	453,192	300,441	25,135	7.37%	-20.05%	1.74	0.36	49.98	52.08	95.05	2679.64	49.45	37,850
NC300-KPS-SCL8	466,313	320,886	27,083	7.76%	-15.86%	1.82	0.37	49.98	52.18	95.22	3394.89	52.62	48,022
NDC100-KPS-SCL8	365,338	202,191	16,474	5.33%	-42.16%	1.31	0.26	50.96	52.53	95.87	1286.10	34.07	17,738
NCP1-KP-SCL1	469,461	326,516	27,632	7.86%	-14.76%	1.84	0.38	3.68	5.90	10.76	246.35	53.48	3,487
NCP1-KPS-SCL1	237,977	126,465	10,314	3.36%	-63.58%	0.84	0.15	7.12	8.12	14.81	95.04	21.46	**1,255**
NCP3-KP-SCL1	456,250	312,014	26,390	7.56%	-18.03%	1.78	0.36	3.65	5.78	10.55	194.76	51.22	2,730
NCP3-KPS-SCL1	264,102	148,987	12,191	3.86%	-58.08%	0.96	0.17	6.84	7.97	14.55	101.32	25.04	**1,340**
NCP3-KPS-SCL2	73,584	41,560	3,483	1.05%	-88.58%	0.27	0.04	9.60	9.90	18.07	90.11	7.01	**1,145**
NCP5-KP-SCL1	465,627	325,336	27,684	7.81%	-15.25%	1.83	0.37	3.61	5.80	10.59	231.77	53.30	3,259
NCP5-KPS-SCL1	328,219	195,122	16,041	4.97%	-46.04%	1.21	0.23	6.88	8.31	15.17	135.92	32.55	1,823
NCP10-KP-SCL8	239,530	157,227	13,496	3.80%	-58.82%	0.92	0.16	18.90	19.98	36.47	321.53	26.01	4,187
NCP10-KPS-SCL2	306,458	184,103	15,133	4.66%	-49.41%	1.14	0.20	12.91	14.24	26.00	256.82	30.67	3,416

All of the intervention scenarios reduced the expected number of infected people (children, adults, and seniors) and the expected number of influenza deaths compared to the baseline. As a result, both the medical costs associated with illness and the costs associated with staying at home with sick children were reduced. The additional costs associated with the interventions are the costs of parents and children staying home due to school closure. Taking NCP3-KPS-SCL2 as an example, both the medical costs associated with illness and the cost associated with staying home with sick children were reduced by 80% compared to the baseline, and the cost per case prevented by the intervention was USD1,145. Although this was not the cheapest school closure strategy, it achieved the best effectiveness in terms of preventing infected cases—it resulted in an 80% decrease in patients, which is equivalent to the prevention of about 830,000 cases in a city of 7 million people.

This study demonstrated that the effects of individual school closure strategies depend on (i) school types closed, (ii) closure modes, (iii) closure triggers, and (iv) closure lengths, particularly for individual school closure strategies. Perlroth et al. [19] commented that although school closure is an effective method for containing the spread of infectious diseases, it has substantially higher costs than the “do nothing” strategy. In this study, we confirmed that all of the school closure strategies resulted in higher costs than the baseline, unmitigated strategy. However, we also demonstrated that the cost of an intervention can be greatly reduced by replacing traditional school closure methods with individual closures based on a carefully selected trigger. Some studies have suggested that early school closures are often associated with great reductions in peak and cumulative ARs [49]. Our study showed that closing schools based on the number of confirmed cases at the school, if small thresholds are used, leads to the greatest reductions in peak and cumulative ARs and are the most economically viable strategies. We predicted that the effect of school closures increased with the duration of closure; but for scenarios that closed kindergartens only, this effect was minimal. Wu et al. [22] demonstrated a significant reduction in original transmission due to the special schooling arrangements in place in Hong Kong. Our contribution here is to provide a model of the predicted effects of a variety of individual school closure strategies based on the H1N1 pandemic.

We made several assumptions in this study. First, school vacations or seasonality were ignored within the simulation timeframe. Second, we assumed that school closures would not increase the per-contact probabilities of other social contact groups, such as family, household, and community. In fact, students may increase visits to public venues during school closures [9] and may increase contact between household members if they stay at home. Future studies should examine the effects of the closure of particular grades or classes (with the remainder of the school open) [2]. The potential challenge is that measuring the effects of grade/class dismissal would require a more-detailed community-based dataset at the school class level. Further research could also consider optimal strategies that combined various system-wide and district-wide school closures with individual school triggers across types of schools, and varying closure triggers during different phases of the pandemic, as well as the synergistic effect of combining effective school closure strategies in conjunction with other pharmaceutical interventions.

There were scattered school suspensions throughout the HK H1N1 pandemic. We examined various strategies based on realistic scenarios that were feasible, potentially implementable, and practical. Our study provided cost-effectiveness analyses of various individual-based school closure scenarios and attempted to balance the epidemiologic and economic benefits of such interventions. Public health policy makers have demonstrated the ability to identify the number of confirmed infectious cases in schools [2]. We foresee that implementation of an individual school closure policy would not be too difficult. Since decision makers will be provided with specific rules-based closing strategies, it would be reasonably feasible for the decision makers to identify situations when the rules call for the closing of a particular school. Furthermore, closings will not necessarily come as a surprise since warnings could be given to parents stating that school closures are imminent if the disease count at a particular school is approaching the level at which school closure will be triggered. However, previous national and local experiences with school closures also tell us that decisions about school closure are not trivial. Substantial communication, cooperation, and coordination among authorities, the public, and schools would be needed to enable effective future implementations, and these must be based on sound scientific proof of appropriate triggers and lengths for given national/regional demographic populations.

## Conclusions

This paper evaluated via simulation the cost-effectiveness of various individual-based school closure intervention strategies. The estimated cost incurred in the 2009 Hong Kong H1N1 pandemic of USD330 million is used as a baseline. Our study revealed that closing individual schools based on the number of confirmed cases at the schools using small thresholds, leads to the greatest reduction in peak and cumulative ARs and is the most economically viable strategy. Such strategies could result in an 80% decrease in cases, preventing about 830,000 infections; the cost per case prevented due to intervention was estimated as USD1,145.

## Supporting Information

S1 File Details on Economic Evaluation Parameters and Probabilistic Sensitivity Analysis. Age-stratified healthcare decision model (**Table A**). Cost analysis model parameters (HKD) (**Table B**). Probability distributions of critical cost parameters (**Table C**). Probabilistic sensitivity analysis (NCP3_KPS_SCL2 vs. baseline) (**Figure A**). Probabilistic sensitivity analysis (FCF2_KPS_SCL2 vs. baseline) (**Figure B**). Probabilistic sensitivity analysis (NC300_KPS_SCL8 vs. baseline) (**Figure C**). Probabilistic sensitivity analysis (NDC100_KPS_SCL1 vs. baseline) (**Figure D**).
(DOCX)
