# Supplementary material for: Economic Evaluation of Individual School Closure Strategies: The Hong Kong 2009 H1N1 Pandemic
Source: PLoS One. 2016 Jan 28;11(1):e0147052. doi: 10.1371/journal.pone.0147052 (PMC4731466; doi:10.1371/journal.pone.0147052)
Supplement: S1 File — Age-stratified healthcare decision model (Table A). Cost analysis model parameters (HKD) (Table B). Probability distributions of critical cost parameters (Table C). Probabilistic sensitivity analysis (NCP3_KPS_SCL2 vs. baseline) (Figure A). Probabilistic sensitivity analysis (FCF2_KPS_SCL2 vs. baseline) (Figure B). Probabilistic sensitivity analysis (NC300_KPS_SCL8 vs. baseline) (Figure C). Probabilistic sensitivity analysis (NDC100_KPS_SCL1 vs. baseline) (Figure D). (DOCX) [file pone.0147052.s001.docx]

**S1 File. Details on Economic Evaluation Parameters and Probabilistic Sensitivity Analysis**

**Economic model**

This document provides details with regard to additional work on the cost parameters used in the cost analysis model. In particular, based on the best scenarios of each school closure strategy, we present probability sensitivity analysis results considering the uncertainty of key cost parameters.

**Age-group stratified health outcomes**

The total cost of each intervention scenario (including the baseline) was the sum of the medical costs associated with the illness, the costs associated with parents staying at home with sick children, and other costs associated with school teachers, parents, and children staying home due to school closures. Referring to [1, 2], the age-group stratified health outcomes related to influenza can be characterized with respect to children (0-18), adults (19-59), and seniors (60+). The health outcomes include outpatient visits, prescription and over-the-counter (OTC) drugs, hospitalisation, and lost earnings due to death [1, 2]. Wu et al. 2010 [3] provided the age-stratified case-hospitalization and case-fatality rates in Hong Kong during the 2009 H1N1 pandemic. The case-hospitalisation rates in the 2009 influenza pandemic ranged from 0.47% to 0.87% among people aged 5 to 59 years; the age-group-specific case-fatality rates ranged from 0.4 to 26.5 cases per 100,000 infections in people aged 5 to 59 years. We interpolated the hospitalisation rate and death rate for children based on the reported results and number of children in the age groups 5-14 and 15-19 in the simulation model. A similar method was applied when we calculated the hospitalisation and death rates for adults. Since the study did not report hospitalisation and death rates for seniors (60+), we assumed the reported case-hospitalisation and case-fatality rates for ages 50-59 and the senior group (60+) are the same. Combining this with the cost structure suggested by Meltzer et al. [2] and Andradóttir et al. [1], we obtain the age-stratified healthcare decision model for the Hong Kong outbreak illustrated in Table A. The outpatient visits rates were directly referred to in [1], and from those we deduced the rates of the ill but no medical care sought components.

**Table A. Age-stratified healthcare decision model.**

|  | **Probability of Outpatient Visit [1, 2]** | **Probability of Hospitalisation (**$\boldsymbol{\times}$**10^-3^) [3]** | **Probability of Death (**$\boldsymbol{\times}$**10^-4^) [3]** | **Probability of Ill but No Medical Care**  **Sought** |
| --- | --- | --- | --- | --- |
| Children | 0.165 | 8.100 | 0.150 | 0.827 |
| Adults | 0.040 | 6.660 | 1.200 | 0.953 |
| Seniors | 0.045 | 8.700 | 2.650 | 0.946 |

Table B provides the cost estimates and medical price indices drawn from various sources in Hong Kong [4-8] and global studies [1, 2, 9]. The formula for each cost item, major assumptions used, and relevant references related to the computation are displayed under the column “Formula/Details from References [1, 2, 4-12]”.

**Table B . Cost analysis model parameters (HKD).**

| **Outcome Category Item** | **Children**  (0-18) | **Adults**  (19-59) | **Seniors** (60+) | **Formula/Details from References [1, 2, 4-12]** |
| --- | --- | --- | --- | --- |
| **Outpatient Visits** |  |  |  |  |
| Average no. visits per case [1, 2] | 1.52 | 1.52 | 1.52 | Obtained from Table 7 of [1] |
| Net payment per visit [1, 2, 5, 6] | 1,391 | 1,079 | 1,419 | Net payment per visit for Adults is computed based on  $Net payment=\frac{Total expenditure-Private household out of pocket expenditure}{Number of vists}$  We obtained private household out of pocket expenditures from [5] and the number of visits from [6]. Values for Children and Seniors were inferred using appropriate ratios derived from Table 7 of [1]. |
| Avg. co-payment per visit [4] | 100 | 100 | 100 | Obtained from [4]. |
| Net payment per prescription [1, 2, 10] | 10.8 | 15.5 | 15.5 | Based on Table 18 (outpatients) of [10], we computed net payment per prescription for Seniors (i.e., HKD 321,570/20,804 = 15.5 / prescription). Values for Children and Adults were inferred using appropriate ratios derived from Table 7 of [1]. |
| Avg. co-payment per prescription [1, 2, 10] | 106.9 | 153.9 | 153.9 | Based on Table 18 (outpatients) of [10], we computed average co-payment per prescription for Seniors (i.e., HKD 3,201,730/20,804 = 153.9 / prescription). Values for Children and Adults were inferred using appropriate ratios derived from Table 7 of [1]. |
| Avg. prescriptions per visit [1, 2] | 0.9 | 1.8 | 1.4 | Obtained from Table 7 of [1]. |
| Days lost [1, 2] | 3 | 2 | 5 | Obtained from Table 7 of [1]. |
| Value of one day lost [1, 2, 7, 11] | 585.2 | 638.4 | 585.2 | Based on Table 2 (outpatients) of [11], we computed the value of one day lost for Adults using:  $Value 1 day lost=\frac{Hong Kong GDP per capita}{365}$  where HK GDP per capita in 2009 is given in [7]. For the value of one day lost for Seniors, we referred to Table 2 of [11], which provides healthcare outcomes of Senior influenza cases. We computed the value of one day lost for Seniors using the product of (i) the value of work time lost per worker aged 65-74 (HKD 912) and (ii) the relative number of annual flu cases among age group 65-74. Similar to Table 7 of [1], we assumed that the values of one day lost for Children and Seniors are the same. |
| Subtotal | 4,202.2 | 3,329.1 | 5,494.5 |  |
|  |  |  |  |  |
| **Hospitalisation** |  |  |  |  |
| Hospital costs [1, 2, 10] | 9,812 | 20,017 | 32,352 | Based on Table 18 (hospitalization due to pneumonia and influenza) of [10], hospital costs for Seniors are computed using (i) the government cost of influenza for all aged over 65 in HKD and (ii) the number of cases (i.e., HKD 22,128,771/684 = HKD 32,352). Values for Children and Adults were inferred using appropriate ratios derived from Table 7 of [1]. |
| Net payment per outpatient visit [1, 2] | 2,100 | 2,669 | 2,895 | Ratios of net payment per outpatient visit to net payment per visit are obtained from Table 7 of [1]. |
| Avg. co-payment for outpatient visit [4] | 100 | 100 | 100 | Obtained from [4]. |
| Most likely no. of days lost [1, 2] | 5 | 8 | 10 | Obtained from Table 7 of [1]. |
| Value of one day lost [1, 2, 7, 11] | 585.2 | 638.4 | 585.2 | As described above. |
| Subtotal | 14,938 | 27,893 | 41,199 |  |
|  |  |  |  |  |
| **Deaths** |  |  |  |  |
| Average age [1, 2] | 9 | 35 | 74 | Obtained from Table 7 of [1]. |
| Present Value of earnings lost [1, 2, 8, 9] | 2,721,253 | 2,779,026 | 176,319 | $PV earnings lost= Average wage\times\sum_{i=1}^{Average retirement age-median death age (9)} \left( \frac{1+growth rate}{1+discount rate} \right)^{i}$  and  $Average wage=Median houly wage \times Median weekly hours of work\times52$  We obtained the median hourly wage and median weekly hours of work from [8]. The growth and discount rates are from [2], and the average retirement age and median death age are from [9]. Values for Children and Seniors were inferred using appropriate ratios derived from Table 7 of [1]. |
| Most likely hospital costs [1, 2] | 11,480 | 25,304 | 39,209 | Relative ratios of “most likely hospital costs” to “hospital costs” are obtained from Table 7 of [1]. |
| Subtotal | 2,732,733 | 2,804,330 | 215,528 |  |
|  |  |  |  |  |
| **Ill but no medical care sought** |  |  |  |  |
| Days lost [1, 2] | 3 | 2 | 5 | Obtained from Table 7 of [1]. |
| Over-the-counter drugs [1, 2, 11, 12] | 71.9 | 71.9 | 71.9 | Based on Table 18 (medicine costs) of [10], OTC drug costs for seniors are computed using (i) the personal cost in HKD of self-care for influenza for persons aged 65+ and (ii) the number of cases (i.e., HKD 1,676,388/23,327 = 71.9). We assumed that such costs are the same among Children, Adults, and Seniors. |
| Value of one day lost [1, 2, 7, 10] | 585.2 | 638.4 | 585.2 | As described above. |
| Subtotal | 1,827.5 | 1,348.7 | 2,997.9 |  |
|  |  |  |  |  |
| **Staying at home** |  |  |  |  |
| Value of one day lost [1, 2, 7, 10] | 585.2 | 638.4 | 585.2 | As described above. |

**Probabilistic sensitivity analysis of model parameters**

The objective of this study is to assess the cost-effectiveness of different school closure strategies. Apart from the sensitivity analysis reported in the main text in Sections 2.5 and 3.3, we also considered the uncertainty of the cost parameters using probabilistic sensitivity analysis (PSA). PSA is a procedure that considers input parameters as random quantities, and associates with each parameter a probability distribution that describes its state as in a Bayesian framework [13]. The previous section in this appendix described how we calculate all of the cost parameter values. Some of the parameters can be represented as probability distributions thanks to information from additional sources. Table C depicts the distributions of two critical cost parameters: (i) the value of one day lost for adults and (ii) the most likely number of days lost for hospitalization for elderly patients. Let us consider (i) first. Based on [7], we assumed the Hong Kong GDP per capita over the period 2006-2015 is normally distributed and calculated the (simplistic) sample mean and standard deviation (SD) of (i) naively assuming that the 10 yearly GDP per capita figures over the time of interest were independent and identically distributed. This cost parameter is heavily used when computing the cost components associated with hospitalized sick adults and adults staying at home to look after non-sick child due to school closure. Regarding (ii), Zhou et al. [14] report that the median and interquartile range (IQR) number of days of influenza-related hospitalities are 14 and 23 days, respectively, and are significantly higher than other age groups. A lognormal probability distribution has been used to model such data, and the mean and standard deviation of a lognormal can be estimated based on a simple formula, e.g., [15].

We considered the potential variabilities of factors (i) and (ii) on the best scenarios from different sets of school closure strategies (including KCP3_KPS_SCL2, FCF2_KPS_SCL2, NC300_KPS_SCL8, and NDC100_KPS_SCL1). PSA in health economics analysis using a Bayesian approach has been discussed in some state-of-the-art studies. Based on the statistical framework provided by Baio and Dawid [13], we performed and reported cost-effectiveness plane, cost-effectiveness acceptability, expected incremental benefit, and expected value of information analyses; and these are provided in this appendix. When developing the Bayesian cost effectiveness analysis, we also referred to the comprehensive decision analytical model provided by Cooper et al. [16] and the Bayesian random effects meta-analysis model specifications provided by [17]. R version 3.2.2 under a Windows 64-bit platform and various R packages (namely, R2jags, BECA, ggplots, and R2WinBUGS) were used to conduct the study.

**Table C. Probability distributions of critical cost parameters.**

| **Cost parameters** | **Values** | **References** |
| --- | --- | --- |
| *Value of one day lost for Adults | Mean: 625.44; SD: 82.7 (normal distribution) | HK GDP per capita is given in [7]. Based on 2006-2015 GDP per capita, we calculate the mean and SD of one day lost for Adults. |
| #Most likely no. of days lost (hospitalised) for Seniors | Median: 14; IQR: 23 (lognormal distribution) | According to [14], these values are significantly higher than for other age groups. We reference [15] to estimate the mean and variance of a lognormal distribution of length of stay using the median and IQR. |

*Used when estimating hospitalisation cost for adults and adults staying at home looking after children.

#Influenza related number of days lost is defined as the length of the first hospital admission after the onset of influenza symptoms.

**PSA results**

We carried out PSA to study the uncertainties associated with the various cost parameters in each “family” of school closure strategies. We considered a set of interventions based on the best scenarios for each school closure strategy (including KCP3_KPS_SCL2, FCF2_KPS_SCL2, NC300_KPS_SCL8, and NDC100_KPS_SCL1) and the baseline scenario. Figures A to D depict the cost-effectiveness plane, cost-effectiveness acceptability, expected incremental benefit, and expected value of information curves corresponding to the four PSA for “best” vs. baseline comparisons.

Here we focus on the discussion and interpretation of the findings for NCP3_KPS_SCL2 vs. the baseline (Figure A), as this is shown to be the most cost-effective scenario. The cost-effectiveness plane on the top left-hand side reports the Incremental Cost Effectiveness Ratio (ICER), which is defined as the expectation of the difference in the two scenarios’ mean costs divided by the expectation of the increment in mean effectiveness, i.e., E[∆c]/E[∆e]. Cost refers to the associated economic cost described previously and effectiveness refers to the number of influenza cases associated with each intervention. While the expectation is over the subjective distribution of the cost parameters, the resulting ICER is reported as USD93.02 (this value is also the smallest among the other school closure strategies shown in Figs 3 and 4 of the main text). Initial burn-in runs of 5000 iterations were used and discarded. In order to achieve convergence [16], inferences based on a further 10,000 iterations were computed. The results lie on the plane indicating that the individual school closure scenario is more effective compared to the baseline but more costly.

The expected incremental benefit (EIB) and the accompanying 95% credible intervals are shown in the top right-hand corner. EIB is defined as k*E[∆e]- E[∆c], where k is a “willingness-to-pay” value (unit: USD). It can be shown that for k < 160, the baseline is the more cost-effective option (i.e., EIB < 0). However, if we are willing to invest a value exceeding k, the individual school closure scenario becomes cost-effective.

It is common to summarize the PSA results using a cost-effectiveness acceptability curve (CEAC). This provides a simple summary of the probability of cost-effectiveness upon varying the k parameter. Varying the parameter k from 0 to 40,000, we can see the probability of cost-effectiveness is high (shown in the bottom left-hand side), indicating a low uncertainty in the actual cost-effectiveness of the individual school closure strategy (NCP3_KPS_SCL2). When we look at other CEAC curves from other scenarios (i.e., FCF2_KPS_SCL2, NC300_KPS_SCL8, and NDC100_KPS_SCL1) for such small values of k, the probabilities of cost-effectiveness is low as 0. This implies that the individual school closure strategy (NCP3_KPS_SCL2) has lower uncertainty in achieving actual cost-effectiveness than other school closure scenarios (i.e., FCF2_KPS_SCL2, NC300_KPS_SCL8, and NDC100_KPS_SCL1).

On the bottle right-hand side, the expected value “perfect” information (EVPI) graph provides an analysis of the individual expected value of information (Vi) as a function of the k parameter. The breakeven point is at k and the shape of the EVPI curve changes because the optimal decision is reversed beyond that point. As shown, the EVPI value fluctuates a great deal after the breakeven point. This indicates that the uncertainties in the parameters may have a significant impact on the result.

**Figure A. Probabilistic sensitivity analysis (NCP3_KPS_SCL2 vs. baseline).**


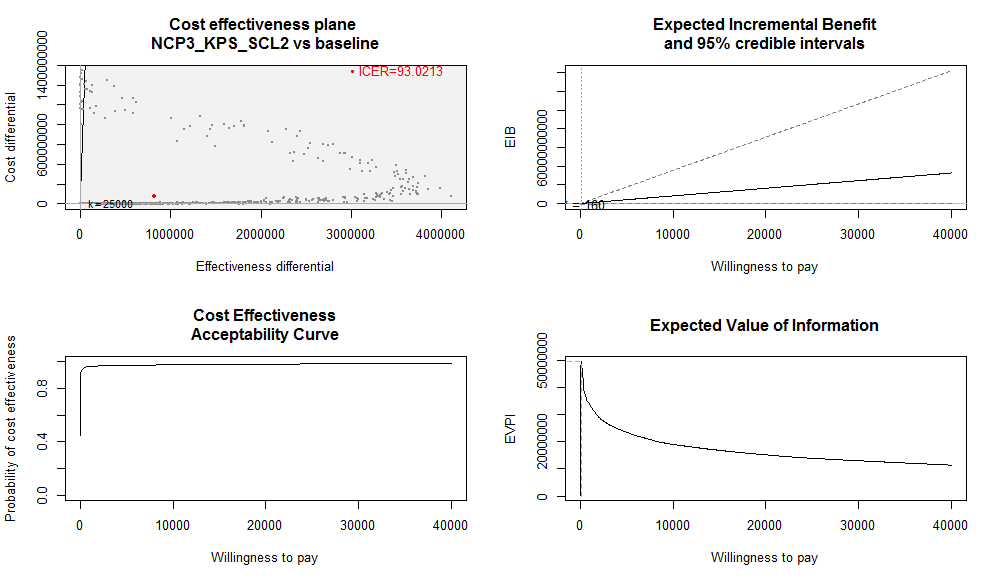


**Figure B. Probabilistic sensitivity analysis (FCF2_KPS_SCL2 vs. baseline).**


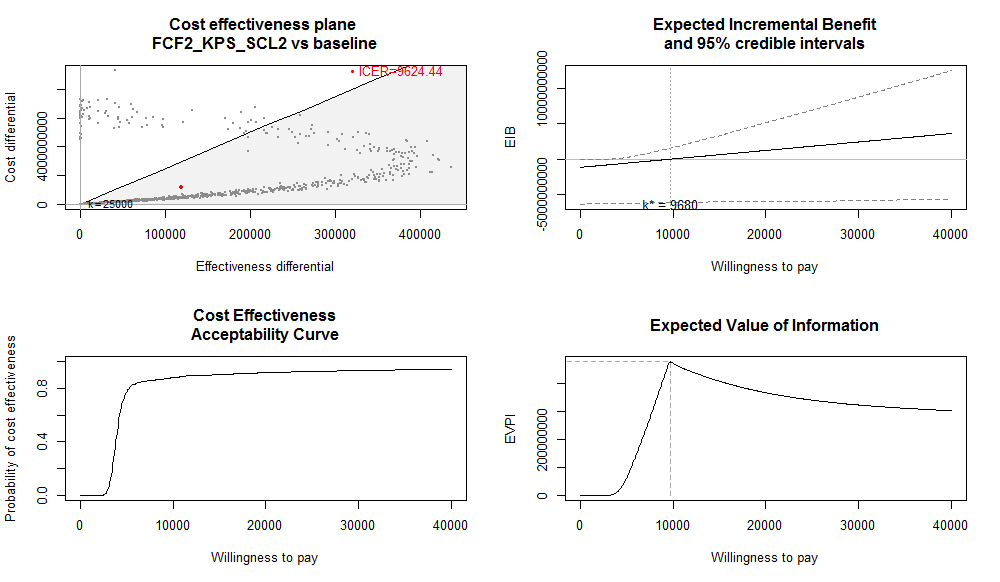


**Figure C. Probabilistic sensitivity analysis (NC300_KPS_SCL8 vs. baseline).**


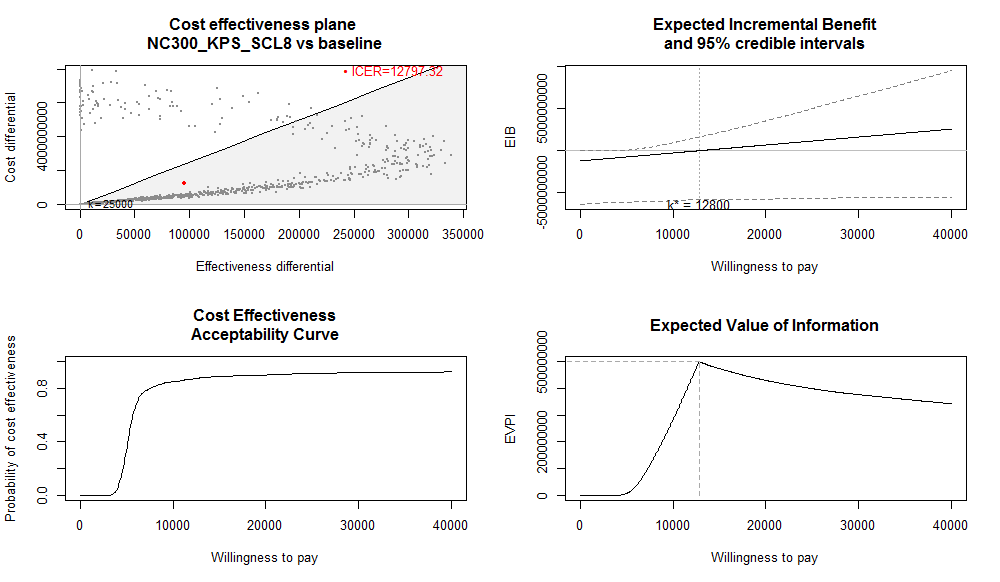


**Figure D. Probabilistic sensitivity analysis (NDC100_KPS_SCL1 vs. baseline).**


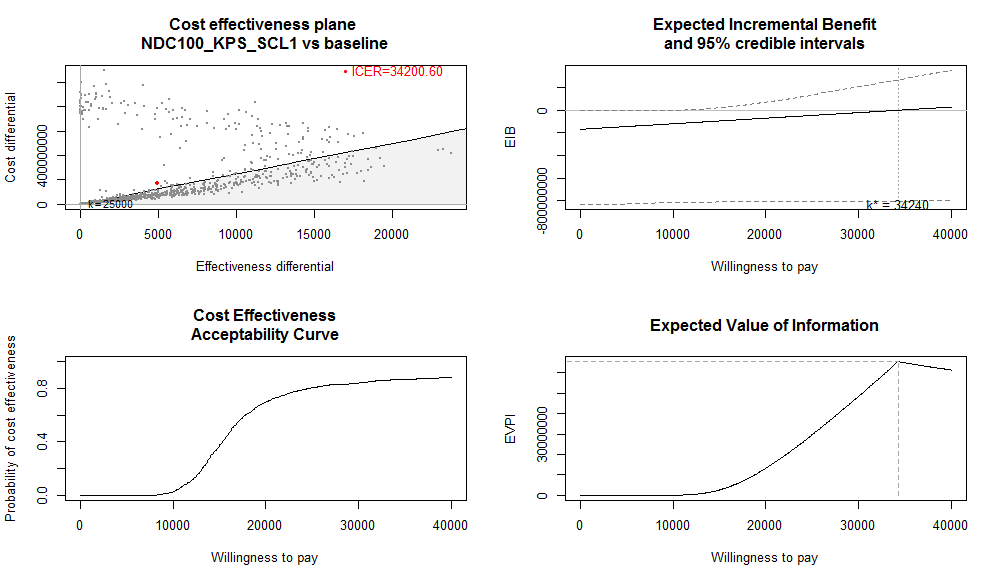


**References**

1. Andradóttir S, Chiu W, Goldsman D, Lee M, Tsui K-L, Sander B, et al. Reactive strategies for containing developing outbreaks of pandemic influenza. BMC public health. 2011;11(Suppl 1):S1.

2. Meltzer MI, Cox NJ, Fukuda K. The economic impact of pandemic influenza in the United States: priorities for intervention. Emerging infectious diseases. 1999;5(5):659-671.

3. Wu JT, Ma ES, Lee CK, Chu DK, Ho PL, Shen AL, et al. The infection attack rate and severity of 2009 pandemic H1N1 influenza in Hong Kong. Clinical infectious diseases : an official publication of the Infectious Diseases Society of America. 2010;51(10):1184-1191.

4. Fees and Charges. Hospital Authority. [cited 2015 May 19]. Available from: <http://www.ha.org.hk/visitor/ha_visitor_index.asp?Content_ID=10045&Lang=ENG&Dimension=100&Parent_ID=10044&Ver=HTML>.

5. Total health expenditure by financing source and function. Food and Health Bureau. 2010 [cited 2015 Apr 11]. Available from: <http://www.fhb.gov.hk/statistics/download/dha/en/tf6_1011.pdf>.

6. Hospital Authority Statistical Report. Hospital Authority. 2011 [cited 2015 May 19]. Available from: <http://www.ha.org.hk/upload/publication_15/321.pdf>.

7. Hong Kong GDP per capita. Trade Economics. 2015 [cited 2015 May 19]. Available from: <http://www.tradingeconomics.com/hong-kong/gdp-per-capita>.

8. Hong Kong Annual Digest of Statistics. Census and Statistics Department. 2010 [cited 2015 May 19]. Available from: <http://www.statistics.gov.hk/pub/B10100032010AN10B0100.pdf>.

9. Average and Median Ages of 2009 H1N1-Related Deaths in the United States, Spring and Fall 2009. CDC. 2010 [cited 2015 May 19]. Available from: <http://www.cdc.gov/h1n1flu/ages_deaths.htm>.

10. McGhee SM, Yeung RYT. Cost-effectiveness analysis of vaccinating the community-living elderly against influenza in Hong Kong. Health and Health Services Research Fund, 2006. Contract No.: Final Report HHSRF # 01030691.

11. Schooling CM, Wong LC, Chau J, Cheung A, Ho A, McGhee SM. Cost-effectiveness of influenza vaccination for elderly people living in the community. Hong Kong medical journal. 2009;15 Suppl 6:44-47.

12. Supermarket Price Watch. Consumer Council 2015 [cited 2015 Oct 12]. Available from: <https://www3.consumer.org.hk/pricewatch/supermarket/index.php?filter1=040&filter2=010&filter3=001&lang=en>.

13. Baio G, Dawid AP. Probabilistic sensitivity analysis in health economics. Statistical methods in medical research. Sept 18, 2011.

14. Zhou L, Situ S, Huang T, Hu S, Wang X, Zhu X, et al. Direct medical cost of influenza-related hospitalizations among severe acute respiratory infections cases in three provinces in China. PloS one. 2013;8(5):e63788.

15. Hozo SP, Djulbegovic B, Hozo I. Estimating the mean and variance from the median, range, and the size of a sample. BMC medical research methodology. 2005;5:13-22.

16. Cooper NJ, Sutton AJ, Abrams KR, Turner D, Wailoo A. Comprehensive decision analytical modelling in economic evaluation: A Bayesian approach. Health economics. 2004;13(3):203-226.

17. Prevost TC, Abrams KR, Jones DR. Hierarchical models in generalized synthesis of evidence: An example based on studies of breast cancer screening. Statistics in medicine. 2000;19(24):3359-3376.
